# Analysis of the Diversity of Substrate Utilisation of Soil Bacteria Exposed to Cd and Earthworm Activity Using Generalised Additive Models

**DOI:** 10.1371/journal.pone.0085057

**Published:** 2014-01-08

**Authors:** Selene Muñiz, Juan Lacarta, María P. Pata, Juan José Jiménez, Enrique Navarro

**Affiliations:** Department of Biodiversity & Conservation and Ecosystem Restoration, Pyrenean Institute of Ecology, Spanish National Research Council, Zaragoza, Spain; Kantonal Hospital St. Gallen, Switzerland

## Abstract

Biolog EcoPlates™ can be used to measure the carbon substrate utilisation patterns of microbial communities. This method results in a community-level physiological profile (CLPP), which yields a very large amount of data that may be difficult to interpret. In this work, we explore a combination of statistical techniques (particularly the use of generalised additive models [GAMs]) to improve the exploitation of CLPP data. The strength of GAMs lies in their ability to address highly non-linear relationships between the response and the set of explanatory variables. We studied the impact of earthworms (*Aporrectodea caliginosa* Savigny 1826) and cadmium (Cd) on the CLPP of soil bacteria. The results indicated that both Cd and earthworms modified the CLPP. GAMs were used to assess time-course changes in the diversity of substrate utilisation (DSU) using the Shannon-Wiener index. GAMs revealed significant differences for all treatments (compared to control -S-). The Cd exposed microbial community presented very high metabolic capacities on a few substrata, resulting in an initial acute decrease of DSU (i.e. intense utilization of a few carbon substrata). After 54 h, and over the next 43 h the increase of the DSU suggest that other taxa, less dominant, reached high numbers in the wells containing sources that are less suitable for the Cd-tolerant taxa. Earthworms were a much more determining factor in explaining time course changes in DSU than Cd. Accordingly, Ew and EwCd soils presented similar trends, regardless the presence of Cd. Moreover, both treatments presented similar number of bacteria and higher than Cd-treated soils. This experimental approach, based on the use of DSU and GAMs allowed for a global and statistically relevant interpretation of the changes in carbon source utilisation, highlighting the key role of earthworms on the protection of microbial communities against the Cd.

## Introduction

### Biolog Ecoplates

Biolog MicroPlates were developed in the late 1980s to assist in the identification of bacterial strains [1]. These 96-well plates contained carbon sources and a tetrazolium violet redox dye that turned purple if inoculated microorganisms utilised these sources. By comparing the obtained carbon substrate utilisation patterns with databases, it was possible to establish a probable identification [2]. Later, microbial ecologists used Biolog plates to investigate patterns at the community level. Therefore, a new plate specifically designed for community analysis and microbial ecological studies was created; this new plate was referred to as the EcoPlate [3]. The EcoPlate contains 31 of the most useful carbon sources for soil community analysis [3], allowing for community-level physiological profiling (CLPP) of heterotrophic bacterial assemblages. This technique has also been widely used to assess the toxicological impacts of different pollutants [4], including different heavy metals [5]–[9].

The use of EcoPlates results in a CLPP, yielding a very large amount of data that may be difficult to interpret. These difficulties are related to the alteration of the original microbial communities due to soil sampling and pre-treatment as well as bacterial extraction [4], [10]. Moreover, the Biolog procedure can be considered a culture method in which the originally inoculated community is altered. The contribution of a certain species population to the colour profile will depend on its culturability in the Biolog wells and on its interactions with other species (differential growth and competition). The tetrazolium dye also introduces some bias in the profile because not all bacteria are able to reduce it [4]. Thus, the Biolog method is more useful for comparing soil microbial communities than for community characterisation [4]. CLPPs provide little insight regarding the function of the community *in situ* unless they are combined with other microbial methods that do not rely on the culturing of the soil microflora [11], [12].

The pattern of positive and negative responses as well as substrate oxidation rate and extent are highly reproducible for simple microbial communities, particularly if the inoculum densities are similar [10]. Nonetheless, data corrections are required if inoculum density is not controlled. A kinetic approach must be used to extract data from the plates to avoid problems related to incubation time. However, there is no consensus regarding which statistics are most appropriate, and the interpretation of their meaning remains unclear [4].

### Earthworms and cadmium (Cd) on soils

Earthworms are key members of the soil macrofauna in temperate soils [13] that directly or indirectly modulate resource availability (quality, quantity, and distribution) for other organisms. Earthworms are considered ecosystem engineers because they modify, maintain, or create habitats [14] as they build soil biostructures, which consist of aggregates and macropores [15]–[17]. The soil zone influenced by earthworm burrowing and casting was first termed the drilosphere by Bouché, 1975 [18], [19], where microbial biomass is greater than in the surrounding soil [20]. In general, the mechanical and biological activities of earthworms favour organic matter mineralisation and humification and stimulate microbial activity [13].

Cd levels in the environment vary widely, and the average natural abundance of this element in the earth's crust varies from 0.1 to 0.5 mg kg^−1^. However, human activities, such as mining, metal smelting, urban and industrial emissions, waste incineration, coal combustion, traffic dust, and particularly the use of phosphate fertilisers and sewage sludges, are quoted as the primary reason for the increase in soil Cd content over the last several years in Europe [21]. As heavy metals cannot be degraded, they tend to accumulate in soils. Their disappearance indicates that they have leached into deep soil layers and transferred to subterranean waters or transported to other locations by runoff [22]. The fate of Cd in soils will depend on a balance between adsorption, leaching, and biological uptake. In most soils, more than 99% of Cd is found in the solid phase, and less than 1% is present in the soil solution [21].

Earthworm species belonging to the three main ecological groups, i.e. epigeics, endogeics and anecics, have been shown to increase the mobility and bioavailability of some heavy metals in soils [23], [24]. As a result, soil ecosystem function can be altered by heavy metal pollution through changes in microbial communities and earthworms in the drilosphere [25]. These changes in microbial community composition may lead to increased heavy metal tolerance in the bacterial community [26], [27]. However, a decrease in microbial diversity also leads to less soil resilience to natural or human-induced perturbations [28]. Thus, studies on the joint effect of earthworm engineering and soil pollution on the bacterial community may help to prevent and remediate human impacts at the ecosystem level.

### Experimental approach: the use of generalised additive models (GAMs)

Our goal is to demonstrate that the joint action of earthworm activity and Cd toxicity induce changes in the bacterial community that are measurable by changes in the CLPP. To this end, we have assessed and measured these changes using a combination of descriptive and multivariate statistics together with GAMs [29], [30]. GAMs are semiparametric extensions of generalised linear models that allow for the flexible modelling of covariate effects, avoiding the limitations of parametric models.

This methodology is appropriate when the relationship between the variables is expected to be complex and not easily fitted by standard linear or non-linear models, which is the case in most biological studies. One of the most frequent uses of GAMs in ecology is the modelling of species distribution and species abundance [31], related to environmental and geographical variables [32], being plant ecology and fisheries the two fields where the use of GAMs have been generalized in the last decade. Studies of air pollution and health are another domain where these models have been widely used as standard methods since its flexibility in modelling spatial and temporal processes [33]. One of the great advantages of these models is its ability to determine the nature of the relationship between the response and explanatory variables rather than assuming a fixed relationship, allowing for fitting complex relationships in a straightforward manner.

## Methods

### Soil and earthworm sampling

The earthworm *Aporrectodea caliginosa* is commonly used in soil ecotoxicological studies [34]. This earthworm is present in the natural soils surrounding our facilities, is easy to culture and sensitive enough against different toxicants, and being a key actor of the matter cycling and soil formation, the results obtained with it are environmentally relevant.

A total of 102 individuals were hand-sorted in an experimental field near the Pyrenean Institute of Ecology (Montañana, Zaragoza) in June 2011. Experimental soils were collected in an experimental field near Jaca (northern Spain). No specific permissions were required for these activities since both experimental fields from which the soil and the earthworms were collected belong to the Spanish National Research Council. The activities neither involve vertebrates nor endangered or protected species.

The soil pH was determined from extracts obtained with a 1:2.5 ratio (g soil:g distilled H_2_O [pH_w_] or in 0.1 M KCl [pH_KCl_]). Organic matter (OM) content, cation exchange capacity (CEC), and soil texture were measured by calcination, atomic absorption spectrometry, and aerometry, respectively [35]. Field capacity (0.3 bar) and wilting point (15 bar) were determined in two pressure chambers [36]. The pH values at the earthworm sampling site were 7.82 (water) and 7.43 (KCl). The prevailing soil conditions (pH, OM content, and CEC) are provided in Table 1. The soil was loamy with 44.2% sand, 38.1% silt, and 17.7% clay. The bulk density of dried and sieved soil was 1,270.83 kg m^−3^. The field capacity was 30.3% (w/w), and the wilting point was 14.5% (w/w).

**Table 1:** Soil pH, organic matter (OM), and cation exchange capacity (CEC) at day 32.

Treatment	pH_w_	pH_KCl_	OM	CEC	[Cd] µg g^−1^	[Cd] mg L^−1^
			%	meq 100 g^−1^	in soils	in pore water
**S**	8.02	7.89	5.33	17.7	< 0.5	-
**Cd**	7.8	7.88	5.28	18.3	329	0.78
**Ew**	7.84	7.91	5.25	19.4	< 0.5	-
**EwCd**	7.63	7.84	5.16	18.7	311±76	0.49±.002

Soil pH was determined in water (pH_w_) and KCl (pH_KCl_). Prior to Cd analysis in soils, six replicates from Cd treatments were integrated, whereas EwCd replicates were analysed separately (accordingly, standard deviation is available).

All earthworms remained in a terrarium with the same soil from which they were collected for two weeks (maintenance period). Then, they were moved to another terrarium that contained the experimental soil sieved at < 2 mm. The duration of this preconditioning period was 16 days, and its purpose was to allow the earthworms to empty their guts of the previously ingested soil.

### Design and duration of the experiment

**Figure 1:**
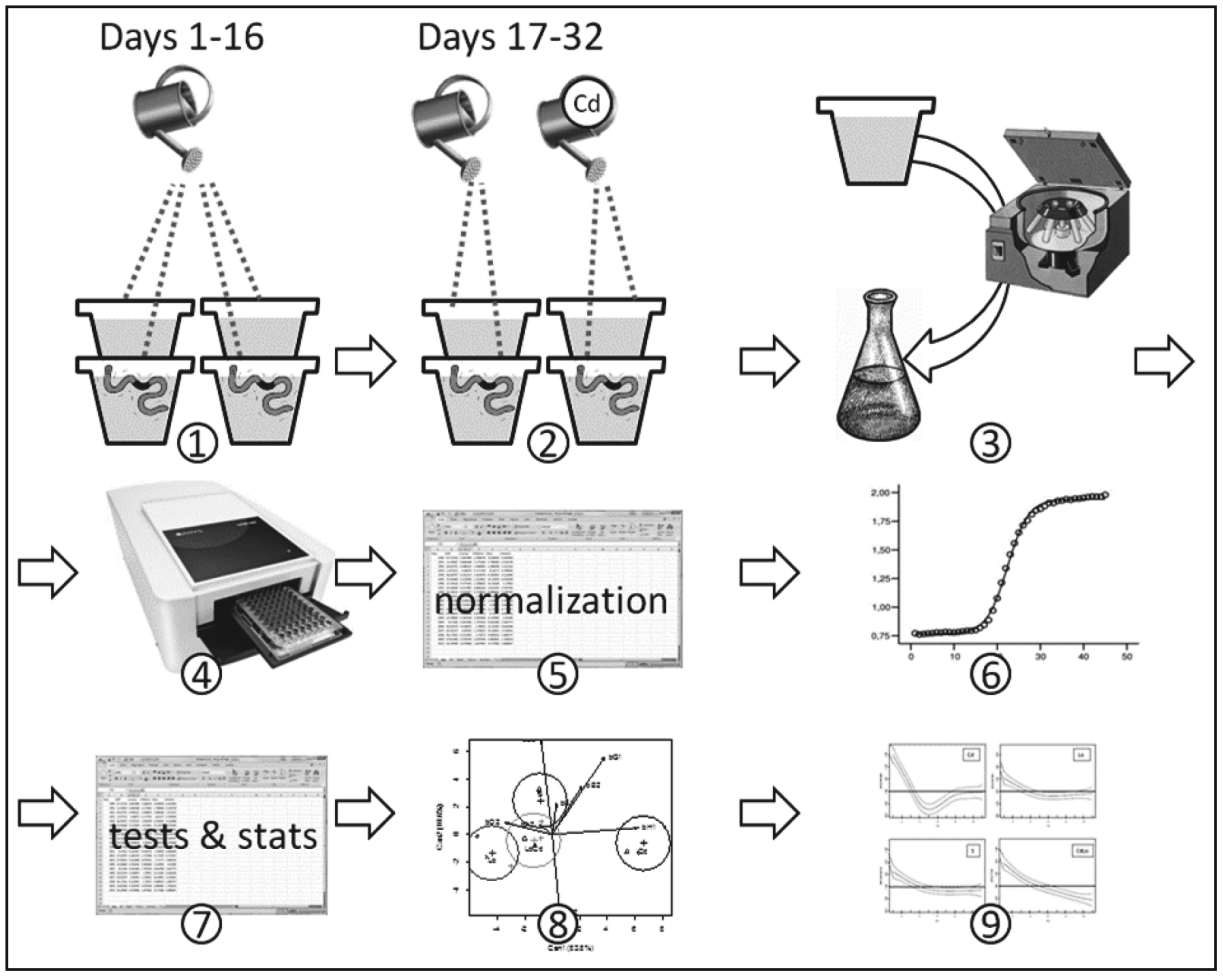
Diagram showing experimental design and data processing. During the first days (1), soil bacteria were exposed to earthworms and irrigated with clean water. Later, soils were irrigated with Cd or clean water depending on the treatment (2). At the end of the experimental period, bacteria were extracted by centrifugation and suspended in liquid media (3). This media was inoculated in Biolog EcoPlates (4). The results of colour development over 167 h were recorded and normalised (5). The results were fitted to a sigmoidal model (6), and their parameters were calculated (slope, time to reach 50% of the maximum colour intensity, and maximum intensity reached). Parameters were compared to assess the effects of the different treatments (7), and the carbon sources that were more informative (accumulating more discriminating power) were selected for multivariate analysis (8). Finally, GAMs were fitted to assess the effect of the different treatments on the use of diverse carbon sources over time, determined by the Shannon-Weaver index (9).

We tested the effect of Cd and earthworms on the ability of bacteria to degrade the 31 different sources of carbon included in Biolog EcoPlates. Accordingly, a bifactorial experiment with four treatments was designed (S: soil control; Cd: soil irrigated with Cd; Ew: soil engineered by earthworms; EwCd: soil engineered by earthworms and irrigated with Cd). A detailed schema of the design is provided in Figure 1. Six replicates (pots) per treatment were filled with 1.5 L of sieved soil. The soil surface exposed to air in each pot was approximately 180 cm^2^. The earthworms were exposed to a natural light/dark cycle from June to August. The air humidity as well as air and soil temperatures were controlled. The soil moisture was measured by a Tektronix 1502C TDR Cable Tester reflectometer (Bracknell, Berkshire, United Kingdom) [37]. After the preconditioning period, specimens of a similar size and weight were selected and introduced into each pot (three individuals). Each pot was subsequently watered with distilled water to reach field capacity (i.e., 30% w/w).

All pots were watered with 200 mL of distilled water from Days 1–16. From Day 17 onward, the pots were watered with 200 mL of distilled water or Cd solution depending on the treatment (Figure 1). Cd was added to the soil as CdCl_2_ + 2.5 H_2_O (Panreac), and the pots were watered with a 343 mg L^−1^ solution. The irrigation was designed to achieve soil concentrations below the median lethal concentration (LC_50_) to *A. caliginosa, which* has been estimated in 540 µg Cd g-1 for a 56-day exposure [38]. The percolated water was collected daily 24 h after the watering that generated it and before watering the pots again. The soils were exposed to both Cd and earthworms for 16 days (see Figure 1).

### Measurement of heavy metal

Following Hobbelen et al. [39], three soil sample types were obtained on Day 32. First, pore water was collected by placing 40 g samples of fresh soil into 50 mL Falcon tubes and centrifuging for 40 min (2,000 g). The supernatants were filtered with a Büchner funnel and cellulose filters (0.45 µm pore size; Sartorius Stedim Biotech) and fixed with 69% HNO_3_ (Hiperpur; Panreac) at a final concentration of 4% (v/v). Next, the soil was air dried. To obtain CaCl_2_ extracts, a 5∶1 mixture (5 g of 0.01 M CaCl_2_:1 g of soil) was prepared and shaken for 2 h at 200 rpm. The extracts were filtered and fixed. Finally, the soil samples were acid-digested in a microwave (Milestone digester) by adding 4 mL of HNO_3_ (PA-ISO 69%) and 1 mL of H_2_O_2_ (33%) to each 0.1 g of dried soil (20 min at 220°C, final temperature). All samples were stored at 4°C until further analysis. The Cd concentration in the soil and percolated water was determined by inductively coupled plasma-optical emission spectrometry (ICP-OES).

### Bacterial community-level physiological profiling (CLPP)

The microbial community population structure and changes in carbon source usage were assessed by live/dead staining and Biolog tests, respectively. Bacterial cells were extracted from soils following Katayama [40] and modified as described in Ikeda [41]. Briefly, 10 g of fresh soil was diluted in 95 mL of Milli-Q water. The samples were homogenised for 15 min, and 10 mL of the soil suspension was pipetted into a 50 mL Falcon tube. The sample was sonicated for 1 min and subsequently centrifuged at 1,000 g for 10 min to obtain 9.5 mL of sample. The remaining 0.5 mL was resuspended in 10 mL and submitted to a new dispersion-centrifuge cycle to obtain additional microbial inocula. This cycle was repeated five times until a 47.5 mL sample was obtained, which was refrigerated at 4°C until analysis.

Bacterial extracts were later filtered using 60 µm cellulose filters to reduce interference during OD measurements (for Biolog). Viable and dead cells (live/dead staining) were stained following the LIVE/DEAD protocol [42]. The cells in 10 randomly selected fields per sample were counted using NIS-ELEMENTS software (Nikon ©). The number of viable cells was calculated as total - dead.

For CLPP, integrated soil samples were used (sampling was performed at different depths). One Biolog^TM^ EcoPlate was inoculated per treatment and incubated at 25°C in a dark chamber. Each plate contained three sets (replicates) of carbon sources, and each plate was filled with samples from one pot. An Anthos 2010 microplate reader (Biochrom) was used to read the absorbance in each well at 590 nm (OD_590_) at even intervals from 0 to 167 h.

### Diversity of substrate utilisation (DSU)

DSU was determined by the Shannon-Weaver index (H), which is one of the most commonly used diversity measures [43], [44]. This index considers both the number of species and their abundance. In this case, the H value describes the ability of the bacterial community to degrade more or fewer types of carbon sources, thus being an index of physiological diversity of the bacterial community. Microbial communities that are able to degrade more substrates or/and to degrade them with similar efficiency would have higher values of H. The index was calculated as follows:
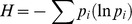



where p_i_ is the normalised value of OD_590_ (see point 2.6). This equation is commonly used to process Biolog data [45], [46].

### Statistical analysis

The carbon degradation data from every well were normalised by being divided by the initial OD_590_ at time 0 after averaging the three wells containing the same carbon source. This procedure was performed to reduce the variability due to differences in inocula densities [47], [48].

The results of colour development showed sigmoidal relationship between the OD_590_ and time for all carbon sources. Therefore, the curves were fitted to a non-linear model [47], [49] with the four-parameter log-logistic function given by the formula:
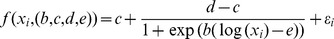



Where parameter b is the slope of the curve (S), *c* is the lower asymptote of the sigmoidal curve, *d* is the upper asymptote of the curve representing the maximal degradation increase (M), and parameter *e* is the time to reach 50% of maximum degradation (TM_50_ or ED_50_). T-tests were performed to evaluate which ratios between parameters from two different treatments were significantly (p<0.05) different from 1; p-values were adjusted using Bonferroni correction for multiple test.

For the CLPP analysis, the carbon sources shown to be more informative regarding the effects of heavy metals on bacterial communities were selected. Other sources (such as D-xylose and D-cellobiose) identified in previous studies were also considered [50]–[52]. Canonical discriminant analysis (CDA) was performed on the selected carbon sources (β-Methyl-D-Glucoside, i-Erythritol, D-Mannitol, α-Cyclodextrin, N-Acetyl-D-Glucosamine, α-D-Lactose, and the two previously mentioned).

Thin-plate regression splines [53] were used as smoothers, with optimal effective degrees of freedom chosen automatically using the generalised cross-validation criteria [30].

All analyses were performed with the free statistical software R [54]. The *drm and compParm* functions of the *drc* package [49] were used to estimate and compare kinetic parameters, the *candisc* package [55] was used for CDA, and the *gam* function of the *mgcv* library [30] was used for GAM modelling.

## Results

### Bacterial cell density and Cd in soils

Cd-treated soils contained significantly fewer bacterial cells (1.1e7 cells g^−1^ DW) than control soils (paired t-tests, p  =  0.03) and a smaller proportion of dead cells (12%). Control soils (S) contained more bacterial cells (4.5e7 cells g^−1^ DW), and earthworm-treated soils had intermediate values (3.0–3.6e7 cells g^−1^ DW). The control and earthworm treatments contained very similar proportions of dead cells (17–20%).

The final Cd concentration in the soils was lower than 0.5 µg g^−1^_dry soil_ in the S and Ew treatments, 328.54 µg g^−1^_dry soil_ in the Cd treatment, and 310.93±75.58 µg g^−1^_dry soil_ (average value, n = 6) in the EwCd treatment. The Cd concentration in the pore water was 0.78 mg L^−1^ in the Cd treatment and 0.49±0.002 mg L^−1^ (n = 2) in the EwCd treatment. The amount of CaCl_2_-extractable metal was 2.18 µg g^−1^_dry soil_ in the Cd treatment and 2.2±0.55 µg g^−1^_dry soil_ (n = 3) in the EwCd treatment.

### Kinetic parameters

The results of colour development revealed sigmoidal relationships between the OD_590_ and time for all carbon sources (see point 6 in Figure 1); thus, the results were adjusted to the four parameters log-logistic model [47]. Three parameters of this model were selected for their usefulness in comparing treatments: the maximum value attained (M), the time at which the colour development is half of its maximum value (TM_50_), and the slope (S). Those parameters presenting significant differences between treatments are shown in Table 2.

**Table 2:** Comparison of kinetic parameters.

Code	Carbon sources	S/Ew	S/Cd	S/EwCd	Cd/Ew	Cd/EwCd	Ew/EwCd
A2	β-Methyl-D-Glucoside				M:1.7	M:2.08	
A3	D-Galactonic acid ∝-Lactone	S:0.26			M:1.61	M:1.86	
		(–0.38,0.90)			(1.46,1.75)	(1.68,2.04)	
B1	Pyruvic Acid Methyl Ester						M:0.78
							(0.68,0.87)
B3	D-Galacturonic Acid		M:0.75		M:1.3		
			(0.67,0.83)		(1.22,1.37)		
C2	i-Erythritol					M:1.59	
						(1.37,1.81)	
C3	2-Hydroxy Benzoic Acid						S:0.14
							(–0.27,0.55)
C4	L-Phenylalanine		S:0.23			M:2	
			(–0.12,0.58)			(1.88,2.12)	
D2	D-Mannitol		M:0.68		M:1.71		
			(0.51,0.85)		(1.56,1.85)		
D3	4-Hydroxy Benzoic Acid	T:0.51	M:0.7			M:1.79	
		(0.12,0.90)	(0.55,0.85)			(1.61,1.97)	
			T:0.55				
			(0.15,0.94)				
D4	L-Serine		M:0.7		M:1.53		
			(0.41,0.99)		(1.42,1.63)		
E1	α-Cyclodextrin					M:1.56	
						(1.38,1.74)	
E2	N-Acetyl-D-Glucosamine		M:0.67		M:1.67		
			(0.41,0.93)		(1.44,1.89)		
F1	Glycogen			M:1.59		M:1.7	
				(1.29,1.89)		(1.50,1.90)	
F2	D-Glucosaminic Acid		T:0.68			M:1.61	
			(0.36,0.98)			(1.36,1.86)	
F3	Itaconic Acid					M:0.75	
						(0.65,0.85)	
F4	Glycyl-L-Glutamic Acid				M:1.68	M:1.72	
					(1.49,1.86)	(1.53,1.91)	
G2	Glucose-1-Phosphate	M:1.53			M:1.36		
		(1.33,1.73)			(1.28,1.43)		
G3	α-Ketobutyric Acid		S:0.27			M:1.6	
			(–0.06,0.60)			(1.50,1.70)	
G4	Phenylethyl-amine	S:0.15					
		(–0.18,0.48)					
H1	α-D-Lactose	S:0.35		S:0.31	S:0.29	S:0.29	
		(–0.06,0.76)		(–0.14,0.76)	(–0.04,0.62)	(–0.32,0.66)	
		M:1.51		M:1.59	M:1.25	M:1.32	
		(1.29,1.73)		(1.35,1.83)	(1.10,1.40)	(1.16,1.48)	
H2	D,L-α-Glycerol Phosphate	M:1.84		M:1.64			
		(1.55,2.13)		(1.33,1.95)			
H3	D-Malic Acid	M:1.65	T:1.93	M:1.5	M:1.72		
		(1.46,1.84)	(1.28,2.57)	(1.31,1.69)	(1.54,2.71)		
H4	Putrescine	S:0.19			M:2.18	M:2.2	
		(–0.20,0.58)			(1.64,2.71)	(1.72,2.68)	

To assess the effect of the different treatments in the degradation of the different carbon sources, the parameters from the 4 parameters log-logistic models, were divided and the resulting ratio compared to 1 using t-tests. The letter indicates the parameter compared (T  =  TM_50_, S  =  slope, M  =  maximum). Values between brackets are 95% CI. Table shows only those comparisons with significant p-values (adjusted using Bonferroni correction for multiple test), meaning that these ratios significantly (p<0.05) different from 1 and thus the parameters compared were significantly different. As example, the first value in the column S/Ew indicates that the slope of the log-logistic model of degradation for A3 in treatment S was four times lower than in treatment Ew.

This process is the first step of the experimental approach proposed in this study; the carbon sources exhibiting significant differences between treatments (i.e., discriminant sources) would be potential candidates for the subsequent data analysis (see Figure 1) with the aim of increasing the discriminant power of the procedure.

Treatments provoked differences in the kinetic parameters of 23 of the 31 carbon sources. Specifically, the largest differences in CLPP were promoted by the activity of earthworms in Cd-treated soils, which accounted for the differences in the kinetics of 13 carbon sources. On the other hand, the addition of Cd to earthworm-treated soils only resulted in differences in two carbon sources (Table 2).

### Canonical discriminant analysis (CDA)

**Figure 2:**
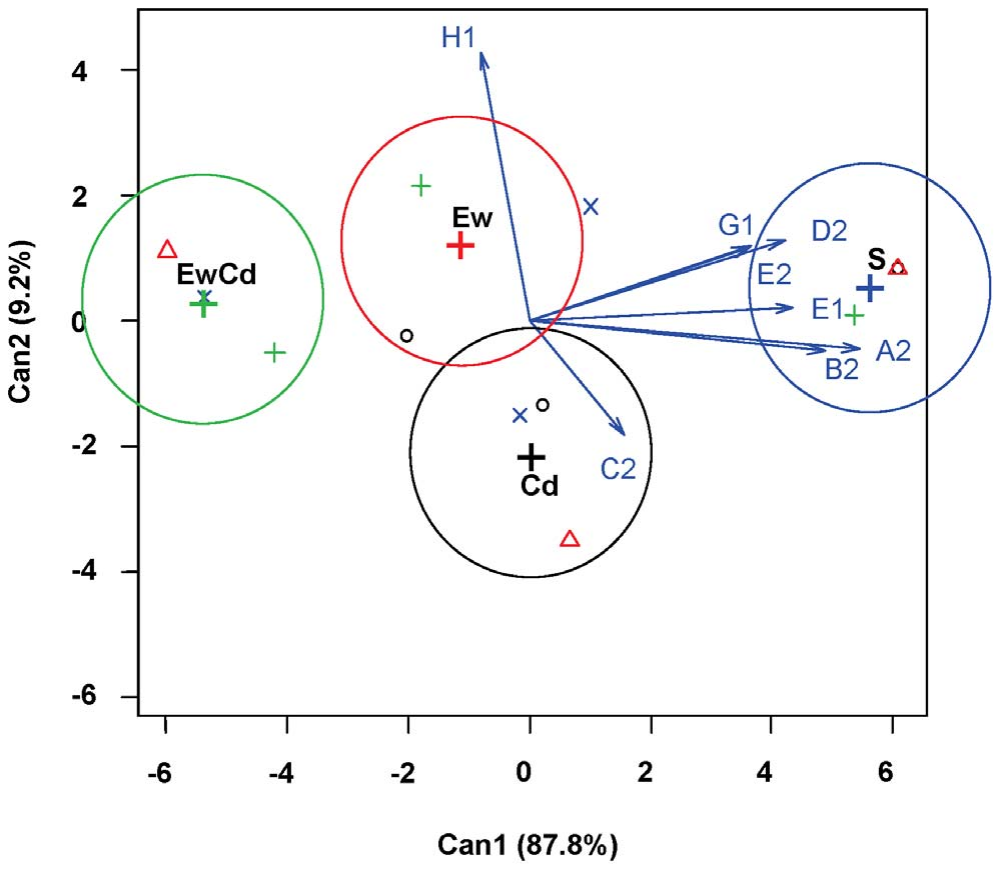
Plot of the two canonical discriminant functions for TM_50_. TM_50_ refers to the time to reach 50% of the maximum colour intensity. Centroid locations of each treatment are indicated by “+”.

**Figure 3:**
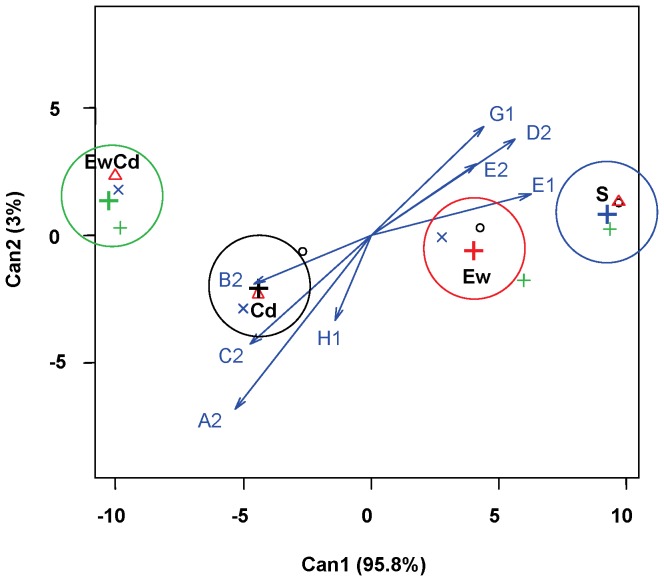
Plot of the two canonical discriminant functions for the maximum colour intensity (M). Centroid locations of each treatment are indicated by “+”.

**Figure 4:**
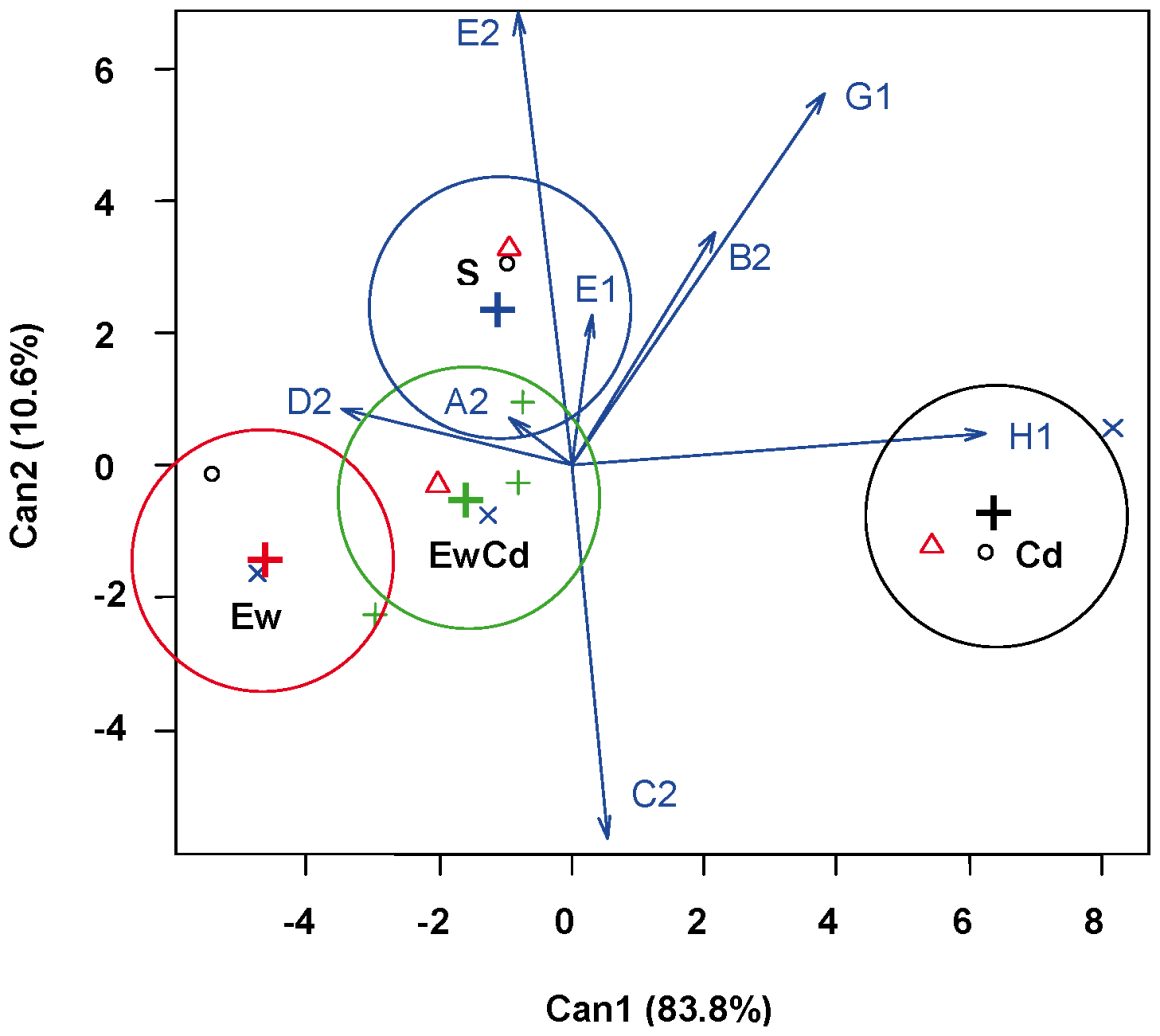
Plot of the two canonical discriminant functions for slope (S). Centroid locations of each treatment are indicated by “+”.

Figure 2 shows the first two CDA axes based on the TM_50_ values of the sigmoidal models (see section 3.1). The cumulative percentage of the variance explained by the first two canonical functions is 97%. The first axis accounts for 87% of the variance and is strongly influenced by D-cellobiose. The second function is strongly influenced by D-cellobiose and is also influenced by N-acetyl-D-glucosamine. Figure 3 presents the CDA based on M values. The cumulative percentage of the first two functions is very high (approximately 99%). In this case, the sources with the most influence are also N-acetyl-D-glucosamine and D-cellobiose for both axes. Figure 4 presents the CDA based on slopes. Although the cumulative percentage is also very high (94%), in this case, the standardised coefficients indicate that the weight of the different sources is more similar than that in the previous CDA.

### Assessing the time course changes in diversity of substrate utilization (DSU) based on the Shannon-Wiener index using generalised additive models (GAMs)

**Figure 5:**
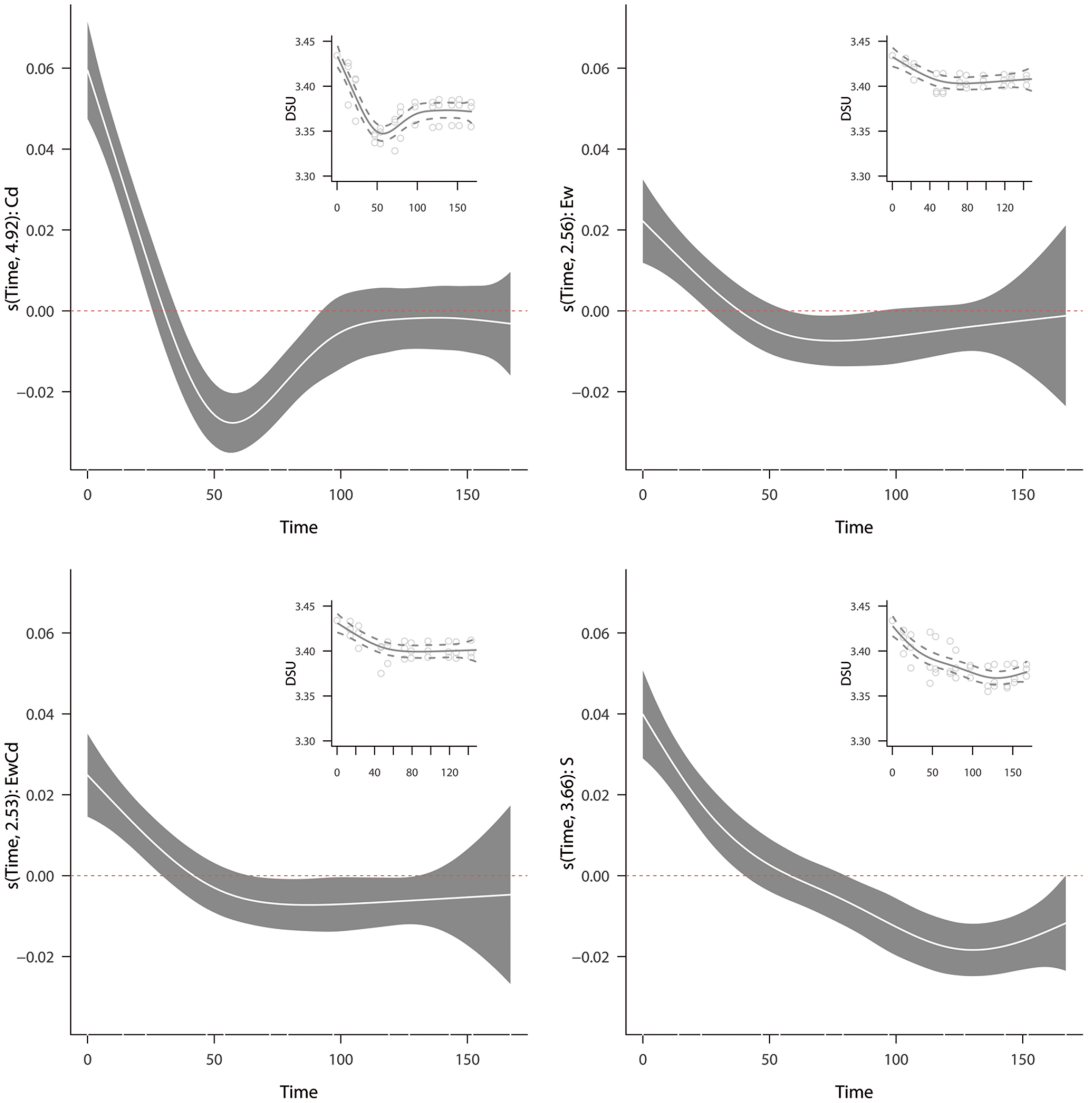
Estimated flexible effects of time-related changes in the diversity of substrate utilisation (DSU). The x axis represents the changes on DSU values over time (hours), obtained from the GAM with the 95% point-wise confidence band (grey coloured) and centred at zero (red dotted line) to allow comparisons. The effective degrees of freedom obtained for each predictor are indicated on the y-axis. The estimated curves in the response scale along with raw data are located in the upper right corner of each plot.

Results from GAM model shows that treatments exhibit significantly different time course changes in DSU, with a explained deviance of 87%. The effective degrees of freedom, *chi* square statistic and p-values of estimated flexible effects from the model are shown in table 3. In Figure 5, the red dotted line represents a hypothetical community with a DSU that was constant as a function of time; in other words, a community exhibiting such a time-course trend would degrade all carbon sources with similar affinity (S) and capacity (M). Thus, this red line can aid in our interpretation of the results. All treatments caused a decrease in DSU during the first 54 h. This decrease was more acute in the bacterial community from the Cd-treated soils. Thereafter, differences were evident between treatments. The communities from soils containing earthworms (Ew and EwCd) presented similar values until the end of the experiment (143 h) regardless the presence of Cd. On the other hand, the community from the Cd-treated soil exhibited an increase in DSU during the next 43 h and did not change thereafter. The community exhibiting the most diverse behaviour was that obtained from control soils (S). In this community, the decline in DSU was constant over time. The model validation did not show any violation of variance homogeneity (i.e. no patterns in residuals) or normality.

**Table 3:** Estimated non-parametric components of GAM model, with the corresponding effective degrees of freedom, F-statistic and P value.

Smooth effect of variable	edf	F-statistic	P value
**f(Time)EwCd**	2.525	8.519	< 0.001
**f(Time)Cd**	4.92	23.868	< 0.001
**f(Time)Ew**	2.555	6.772	< 0.001
**f(Time)S**	3.664	18.483	< 0.001

## Discussion

### Kinetic parameter analysis

Cd exposure resulted in the enhanced degradation of eight carbon sources, as shown by increases in S, M, and/or TM_50_ (see column 2 in Table 2). These results indicate the Cd tolerance of soil microbial communities. It has been shown that long-term exposure to Cd (months to years) may cultivate a more tolerant community [26], [27], exhibiting enhanced enzyme activities [56]. However, our data indicate that the acclimatisation of the communities to Cd may be achieved after only a few days, which has also been shown in communities exposed to mercury [6]. The low percentage of dead cells in Cd-treated soils also supports this observation.

On the other hand, earthworm activity had a modulating effect on Cd impact. Although earthworm activity allowed for the survival of a higher number of bacterial cells, it also caused reductions in bacterial catabolic activity. As shown in column Cd/EwCd in Table 2, earthworm activity resulted in lower M values for 12 carbon sources. Because EwCd contained three times more active bacteria than Cd, this result indicates a marked reduction in their enzymatic activity. The reduction of bacterial growth and activity by earthworms has also been recently described in the absence of toxicants [57].

### Canonical discriminant analysis (CDA)

In all three CDAs (based on TM_50_, M and S), treatments resulted in different CLPPs, as shown by the centroids. The centroids are the weighted position of each treatment in the multidimensional space created by the eight carbon sources used in the analysis (Figs. 2, 3, and 4); arrows indicate the effect of each source in the discriminatory space. The clear separation between centroids means that these sources gathered a sufficient amount of information to differentiate the effect of each treatment on the CLPP. The CDA illustrates how the CLPP is affected by both Cd and earthworms. Compared with Cd, earthworms had a greater effect on the physiological profile of bacterial communities. The three parameters used (TM_50_, M, and S) allow for a clear discrimination between the effects of the four treatments; however, these parameters have different sensitivities for each experimental factor.

#### CDA on TM_50_ (Figure 2)

This parameter allowed for discrimination of the control soils and can be used to detect the interactive effects of Cd and earthworms. The EwCd treatment is characterised by faster degradation of D-cellobiose (G1), α-cyclodextrin (E1), and β-methyl-D-glucoside (A2), whereas Cd is characterised by faster degradation of N-acetyl-D-glucosamine (E2) and D-xylose (B2).

#### CDA on M (Figure 3)

Cd provoked clear differences in M values; its presence reduced the capacity for degrading α-D-lactose (H1), β-methyl-D-glucoside (A2), D-xylose (B2), and i-erytritol (C2), whereas the degradation of D-mannitol (D2), α-cyclodextrin (E1), N-acetyl-D-glucosamine (E2), and D-cellobiose (G1) was enhanced.

#### CDA on S (Figure 4)

Slopes also allow for a clear discrimination of Cd impacts. Thus, the Cd treatment appeared clearly separated from the other treatments. The S values of α-D-lactose (H1) and D-cellobiose (G1) were enhanced by Cd.

#### Sources indicative of specific impacts

The increase in TM_50_ for the degradation of D-cellobiose (code G1 in Biolog plates and in Figure 2) appears to be a promising indicator for earthworm activity. Changes in the slope of α-D-lactose are also indicative of earthworm activity regardless of the presence of Cd. In contrast, an increase in S for 2-hydroxybenzoic acid may be indicative of Cd impacts on bacterial-earthworm consortia.

### Time course changes in diversity of substrate utilization (DSU) by GAM analysis

GAMs revealed significant differences for all treatments (compared to control -S-) in the time trend of DSU. However, all treatments shown a common initial decline (the first 54 h) in the DSU; that is generally attributed to changes in the community inoculated [11], which becomes less diverse. The CLPP would be dominated by fast growing species that are positively selected by high nutrient levels [4]. Other mechanisms related to metal tolerance and earthworm’s effects would explain the differences shown beginning at 54 h and continuing onward.

The acute initial DSU decrease shown by Cd treated soils, suggests the dominance of a few Cd-tolerant microbial taxa [26], [27]. That community presented very high metabolic capacities on a few substrata (as indicated by the results shown in Table 2), and that may explain the acute decrease of DSU (i.e. intense utilization of a few carbon substrata). After 54 h, and over the next 43 h the increase of the DSU suggest that other taxa, less dominant, may reach high numbers in the wells containing sources that are less suitable for the Cd-tolerant taxa.

Earthworms were a much more determining factor in explaining time course changes in DSU than Cd. Accordingly, Ew and EwCd soils presented similar trends and values, regardless the presence of Cd. Moreover, both treatments presented similar number of bacteria (between 3 and 3.6e7 cells g^−1^ DW) and higher than Cd-treated soils (1.1e7). Altogether indicates that earthworm activity provided some protection to the bacterial community against Cd. The bacterial community promoted by earthworms activity during the first 16 days of the experiment, maintained its degrading diversity even after the exposure to a new stressor (Cd). Differently to other studies [58] **,** Cd did not exerted an additional selection pressure. This experimental approach, based on the use of DSU and GAMs allowed for a global and statistically relevant interpretation of the changes in carbon source utilisation, highlighting the key role of earthworms on the protection of microbial communities against the cadmium.
